# A Statistical Analysis of the Sequence and Structure of Thermophilic and Non-Thermophilic Proteins

**DOI:** 10.3390/ijms231710116

**Published:** 2022-09-04

**Authors:** Zahoor Ahmed, Hasan Zulfiqar, Lixia Tang, Hao Lin

**Affiliations:** School of Life Science and Technology, Center for Informational Biology, University of Electronic Science and Technology of China, Chengdu 610054, China

**Keywords:** thermophilic proteins, proteins sequence, secondary structure, hydrogen bonds, salt bridges

## Abstract

Thermophilic proteins have various practical applications in theoretical research and in industry. In recent years, the demand for thermophilic proteins on an industrial scale has been increasing; therefore, the engineering of thermophilic proteins has become a hot direction in the field of protein engineering. However, the exact mechanism of thermostability of proteins is not yet known, for engineering thermophilic proteins knowing the basis of thermostability is necessary. In order to understand the basis of the thermostability in proteins, we have made a statistical analysis of the sequences, secondary structures, hydrogen bonds, salt bridges, DHA (Donor–Hydrogen–Accepter) angles, and bond lengths of ten pairs of thermophilic proteins and their non-thermophilic orthologous. Our findings suggest that polar amino acids contribute to thermostability in proteins by forming hydrogen bonds and salt bridges which provide resistance against protein denaturation. Short bond length and a wider DHA angle provide greater bond stability in thermophilic proteins. Moreover, the increased frequency of aromatic amino acids in thermophilic proteins contributes to thermal stability by forming more aromatic interactions. Additionally, the coil, helix, and loop in the secondary structure also contribute to thermostability.

## 1. Introduction

Proteins are large biomolecules containing one or more long chains of amino acid residues. Enzymes are complex proteins that are involved in life-essential processes like DNA replication, transcription, translation, metabolism, and signal transduction [1,2]. Enzymes can also carry out chemical transformations, which makes them valuable for industrial applications as biocatalysts [3,4]. In the early 2000s, biocatalysts were used for the synthesis or resolution of optically active intermediates [3,5]. Since then, biocatalysts have gradually evolved as applicable tools for the large-scale synthesis and manufacturing of chemicals; thus the demand for biocatalysts is increasing [6,7].

At present, biocatalysts are extensively used in the pharmaceutical, food, animal nutrition, cosmetics, and beverage industries [8,9,10]. In addition, the use of biocatalysts has also entered the detergent, textile, pulp, and paper industries, and into organic synthesis, natural gas conversion, and the biofuel industries [11,12]. Common biocatalysts used on an industrial scale include proteinase and protease for food processing, α-amylase and xylanases in paper bleaching, cellulase and lipase in polymer breakdown, pullulanase as detergents, l-haloacid dehalogenase for chiral halo-carboxylic acid production, and *Sulfolobus solfataricus* γ-Lactamase for the synthesis of the γ-bicyclic lactam, which is an important building block for the anti-HIV compound abacavir [13,14,15,16]. Biocatalysts have more advantages with respect to sustainability, process efficiency, exceptional product selectivity, and lower environmental and physiological toxicity when compared to traditional catalysts [17,18]. Therefore, currently, biocatalysts are preferred to traditional catalysts, but there are still several factors that limit their application on an industrial scale, including biocatalyst stability. In fact, the stability of biocatalysts has received attention from many scholars [18,19,20,21,22]. As we know, a higher temperature can improve the efficiency of enzyme catalysis. However, proteins are temperature-sensitive and denature at high temperatures, which hinders the wide application of enzymes in the industry [17]. Using enzymes with high thermal stability to solve this shortcoming is the key to the application of enzymes on an industrial scale. Therefore, using enzymes in thermophiles is a means to solve the problem [18]. In addition, recent advancements in the protein engineering field have made protein engineering facile and have drawn the interest of researchers to engineer thermostable enzymes for industrial use [23,24,25,26,27,28].

Some microorganisms in nature have been seen to survive in severe environmental and thermodynamic conditions, and their biological growth is most ideal between 50 and 100 °C [29]. The organisms living in such harsh conditions of increased temperatures are generally termed thermophiles. The molecular machinery of the thermophiles is developed to withstand and function at high temperatures [30]. These thermophiles produce proteins that are capable to maintain their structure and activity at high temperatures [31]. The question of how these thermophilic proteins remain stable at such high temperatures has attracted more and more attention. In recent years, researchers have focused on discovering the sequence and structural features of thermophilic proteins. This finding is critical for the theoretical description of the principle behind protein thermal stability [30]. In addition, the discovery of relevant factors also helps to design heat-resistant proteins/enzymes that can meet the requirements of industrial processes. 

We designed this study with a view toward the importance of principles behind the stability of thermophilic proteins at high temperatures. We obtained thermophilic proteins from our previous study on thermophilic proteins [32] and searched for their non-thermophilic orthologous. We preferred thermophilic and non-thermophilic orthologous pairs with the optimum growth temperature (OGT) difference of >20 °C, as the higher OGT difference between a thermophilic protein and its non-thermophilic orthologous could give us clear reasons for thermostability in proteins. Finally, we obtained 10 thermophilic proteins and their non-thermophilic orthologous. The structures of these proteins were obtained from the protein database (PDB), and their sequences, secondary structures, hydrogen bonds, bond lengths, bond angles, and salt bridges were analyzed. In the analysis, we found that the polar amino acids glutamic acid, histidine, lysine, arginine, tyrosine, and aromatic amino acids were slightly more frequent in the thermophilic proteins. Moreover, in the secondary structure, the percentage of the coil, helix, and sheet in thermophilic proteins was higher, while the turn percentage in thermophilic proteins was lower. Subsequently, the number of hydrogen bonds and salt bridges of thermophilic proteins increased. Compared with non-thermophilic proteins, the DHA angle in thermophilic protein was wider and the bond length was shorter. The following sections describe the analysis in detail

## 2. Results and Discussion

To understand the important factors that maintain the thermostability in protein, 10 thermophilic proteins and their non-thermophilic orthologous were collected to investigate the effects of their sequence, secondary structure, hydrogen bond, salt bridge, bond length, bond angle, and aromaticity value on thermal stability in proteins.

**Figure 1:**
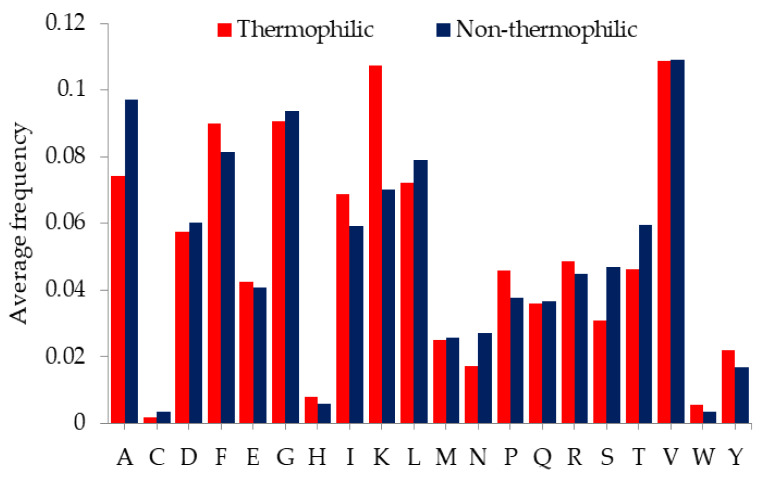
The average frequencies of amino acids in thermophilic proteins and their non-thermophilic orthologous.

The primary amino acid composition (AAC) of a protein imparts specific properties to the protein molecule [32,33,34,35]. Our previous studies have shown that there are significant differences in AAC between thermophilic and non-thermophilic proteins [36,37], which suggests that AAC is the main basis of protein thermostability. Therefore, we analyzed the AAC. Figure 1 shows the frequency of amino acids (AAs) in the thermophilic and non-thermophilic proteins.

As shown in Figure 1, among polar AAs, glutamic acid (E), histidine (H), lysine (K), arginine (R), and tyrosine (Y) have higher frequencies in thermophilic proteins. Polar amino acids R and Y are long-side chain amino acids [38]. Due to their long-side chain, these AAs contribute to the formation of hydrogen bonds, salt bridges, and other long- and short-range interactions to stabilize the protein structure. Moreover, R and Y are abundantly present in the binding hotspot of protein interactions. Hence, it seems that R and Y have similar contributions to the binding and folding of proteins and hinder the unfolding of proteins at elevated temperatures [38,39,40]. In addition, the guanidium group in R involves in the formation of salt bridges that resist thermal denaturation of proteins [38,41,42,43]. Polar amino acid K contains a side chain with a positive charge and forms stable electrostatic interactions with nearby negatively charged groups, and offers stability to the protein structure [44]. It has been reported that amino acid E easily forms interactions in protein to stabilize its structure [45,46,47]. The higher frequency of these polar AAs in thermophilic proteins infers that these AAs contribute to the formation of hydrogen bonds, salt bridges, and other stable interactions to resist the thermal denaturation of proteins at elevated temperatures [48]. Pace et al. [49] also pointed out that the long-range interactions of polar AAs buried in proteins that are not bounded by hydrogen bonding have other interaction forces, such as van der Waals interactions, which stabilize the protein structure. The increased frequency of these polar AAs suggests that they may be the cause to maintain the protein structural stability.

Other polar AAs, including threonine (T), glutamine (Q), asparagine (N), and serine (S), have a lower frequency in thermophilic proteins. Amino acids T and S could interact with water molecules at high temperatures and increase instability in protein molecules [50,51]. Moreover, S has been reported to impair hydrophobic interactions between beta strands [52]. Increased temperature can also cause chemical alterations in AAs. Amino acids Q and N undergo deamidation at elevated temperatures, which imparts an extra negative charge on residues and alters the protein interactions that affect the folding and activity of proteins [53]. The low frequency of these polar AAs in thermophilic proteins suggests that they may be one of the factors that destroy thermal stability in proteins.

Among nonpolar AAs, proline (P) is more common in thermophilic proteins. P has a more rigid structure and plays a role in reducing the entropy of the main chain, resisting the protein unfolding, and stabilizing the loop structure. Due to its hydrophobicity, P interacts with hydrophobic residues on the core and surface of protein molecules, thus preventing the protein from unfolding at elevated temperatures and maintaining protein activity [54]. Isoleucine (I) is also found to be more frequent in thermophilic proteins than in non-thermophilic proteins. Previous studies have also reported the frequent occurrence of I in thermophilic proteins; however, the exact mechanism by which I is used to contribute to thermostability in protein is still not clear [55].

Other nonpolar AAs, including alanine (A), cystine (C), glycine (G), and leucine (L), are less frequent in thermophilic proteins. G and A are amino acids with short-side chains. These AAs form a flexible, rather than a rigid, mechanism. Since their side chains are too short, these AAs form fewer short-range interactions and fail to form long-range interactions to stabilize the protein structure at high temperatures [54]. Amino acid C is easy to deamidate or oxidize at high temperatures, which changes the charge on residues, disturbs the interaction in protein, and affects the folding of the protein [53]. It has been reported that the amino acid L existing in the protein core does not easily form van der Waals and other interactions, resulting in poor thermal stability in the protein [54]. It can be inferred that these unstable amino acids are avoided in thermophilic proteins in order to maintain structural stability and activity at high temperatures. The nonpolar AAs methionine (M) and valine (V) have almost the same frequency in thermophilic and non-thermophilic proteins, indicating that these amino acids have no significant effect on thermostability.

**Figure 2:**
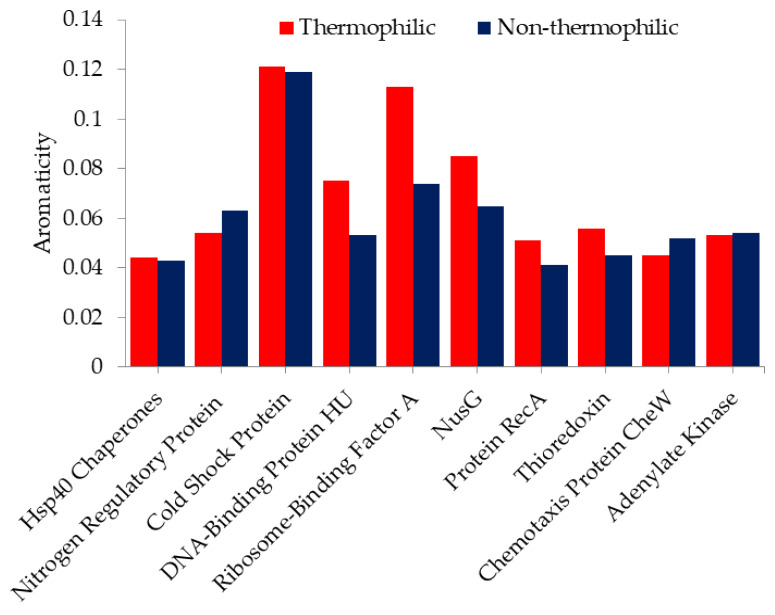
Aromaticity values of thermophilic proteins and non-thermophilic orthologous.

Moreover, we also used aromaticity values to analyze aromatic AAs in thermophilic and non-thermophilic proteins. Aromaticity is the relative frequency of aromatic AAs [56]. Figure 2 shows the aromaticity value of thermophilic proteins and their non-thermophilic orthologous. As the figure shows, except for nitrogen regulatory protein, thioredoxin, and chemotaxis protein CheW, most of the thermophilic proteins have high aromaticity, which indicates that aromatic AAs are preferred in thermophilic proteins. Aromatic AAs form stable aromatic interactions, which contribute to thermal stability. Anderson et al. and Serrano et al. [57,58] have reported that a pair of aromatic interactions contribute 0.5 to 1.4 kcal/mol energy, which means that the increase in aromaticity in thermophilic proteins helps to endow proteins with thermal stability. It is also confirmed by protein engineering methods that the introduction of aromatic clusters on the surface of protein can improve stability in the protein. Kannan et al. [59] have analyzed aromatic clusters in 26 thermophilic proteins and their non-thermophilic orthologous. They found that thermophilic proteins have higher aromatic clusters. These aromatic clusters were able to produce pairwise interactions, which may be crucial to hinder the thermal denaturation of the protein structure.

**Figure 3:**
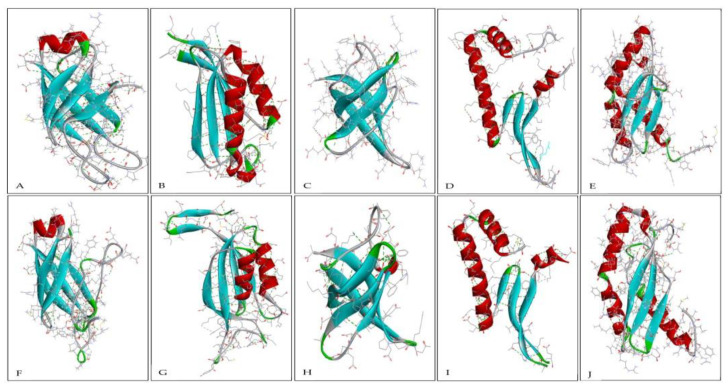
(**A**–**E**) show the structure of thermophilic protein Hsp40 chaperones, nitrogen regulatory protein, cold shock protein, DNA-binding protein HU and ribosome-binding factor A, respectively. (**F**–**J**) shows the structure of their non-thermophilic orthologs, respectively. Coil structure is shown in gray, helix in red, sheet in blue, and turn in green. Conventional hydrogen bonds are represented by the dotted green line, carbon-hydrogen bonds by the light green dotted line, and salt bridges by the dotted yellow line.

**Figure 4:**
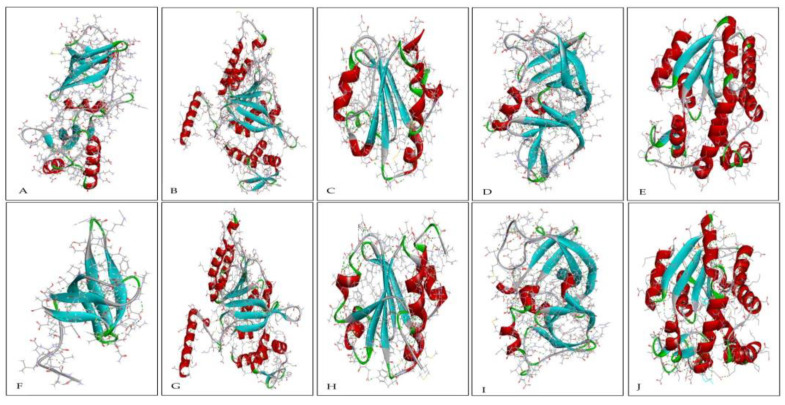
(**A**–**E**) show the structure of thermophilic protein transcription antitermination protein NusG, protein RecA, thioredoxin, chemotaxis protein CheW, and adenylate kinase, respectively. (**F**–**J**) shows the structure of their non-thermophilic orthologs, respectively. Coil structure is shown in gray, helix in red, sheet in blue, and turn in green. Conventional hydrogen bonds are represented by the dotted green line, carbon-hydrogen bonds by the light green dotted line, and salt bridges by the dotted yellow line.

The secondary structure is a folded structure formed by hydrogen bonds between partially positive hydrogen atoms and partially negative nitrogen atoms in the backbone [60,61,62]. Common secondary structure elements include coil, helix, sheet, and turn. The secondary structures of all 10 pairs of proteins are shown in Figure 3 and Figure 4, and their percentage is represented in Table 1.

**Figure 5:**
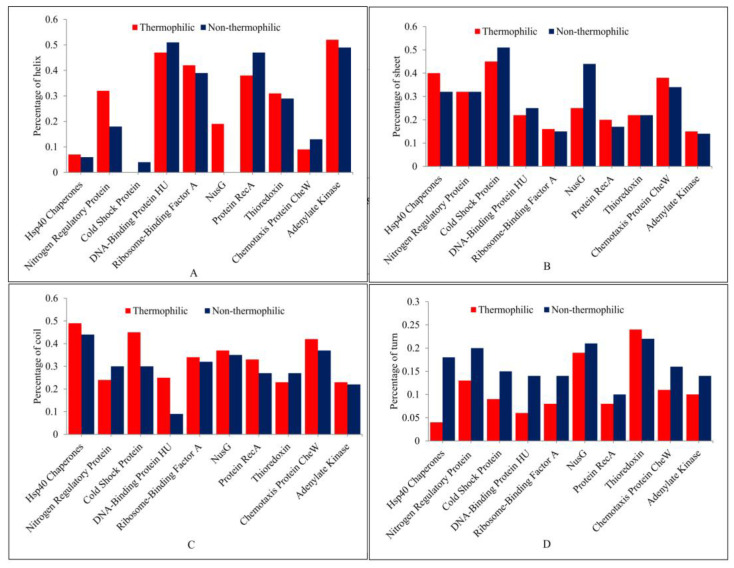
Figure (**A**) shows the percentage of the helix, (**B**) shows the percentage of the sheet, (**C**) shows the percentage of the coil, and (**D**) shows the percentage of the turn structure in thermophilic proteins and their non-thermophilic orthologous.

A helix is a structure formed by hydrogen bonding between every fourth amino acid in a way that makes the side chain of residues directed outward and away from the helical axis, thus allowing the charged residues of the helix to form stable interactions with other elements and resulting in greater stability in the protein structure [63]. The secondary structure analysis showed that most thermophilic proteins, including Hsp40 chaperones, nitrogen regulatory protein, ribosome-binding factor A, transcription antitermination protein NusG, thioredoxin, and adenylate kinase, have a higher helix percentage as compared with their non-thermophilic orthologous (Figure 5A).

Sheet structure consists of two different regions of a polypeptide chain arranged side by side and connected by hydrogen bonds. In our analysis, it was found that the thermophilic proteins Hsp40 chaperones, ribosome-binding factor A, RecA, chemotaxis protein CheW, and adenylate kinase have a higher percentage of sheet structures (Figure 5B). The helix and sheet structures maximize the hydrogen bonding groups of the polypeptide and also allow the protein chains to be buried in the hydrophobic core, making it more compact. The compact protein structure is capable of hindering thermal denaturation [63,64,65]. It is inferred that the increase in the ratio of the helix and sheet structures contributes to thermostability in proteins.

In our analysis, thermophilic proteins have a higher percentage of coil structure than their non-thermophilic orthologous with the exception of nitrogen regulatory protein and thioredoxin (Figure 5C). A higher percentage of coil structure in thermophilic proteins is also reported in the literature [66]. Moreover, the percentage of the turn structure in non-thermophilic proteins except thioredoxin is higher than that of thermophilic proteins (Figure 5D). A turn is considered to allow a change of direction in protein chains. The lower percentage of turns in thermophilic proteins suggests that turns may not be conducive to thermostability in proteins, and the higher percentage of turns in non-thermophilic proteins may contribute to their non-thermostability.

Hydrogen bonds are crucial for the stability of proteins by providing resistance against denaturation [47,64,67]. We analyzed the hydrogen bonds between thermophilic and non-thermophilic proteins (Table 2). The results showed that the thermophilic proteins including nitrogen regulatory protein, DNA-binding protein HU, ribosome-binding factor A, transcription antitermination protein NusG, and protein RecA have a ratio of hydrogen bonds > 0.5. The ratio of hydrogen bonds in Hsp40 chaperones is exactly 5. The increased number of hydrogen bonds in thermophilic protein implies that hydrogen bonds play some roles in protein thermostability. However, in other thermophilic proteins, such as cold shock protein, thioredoxin, CheW, and adenylate kinase, the ratio of hydrogen bonds was <50, indicating that hydrogen bonds are not the key factor in maintaining thermal stability.

Our analysis showed that most of the thermophilic proteins have a higher ratio of salt bridges than their non-thermophilic orthologous. In the present study, the thermophilic proteins Hsp40 chaperones, nitrogen regulatory protein, cold shock protein, DNA-binding protein HU, ribosome-binding factor A, and transcription antitermination protein NusG have a ratio of salt bridges >5, while the other thermophilic proteins including protein RecA, thioredoxin, CheW, and adenylate kinase showed a ratio of salt bridges <5 (Table 2). Salt bridges are electrostatic interactions between oppositely charged groups. Salt bridge is also an important factor contributing to thermostability in proteins. Salt bridges increase thermostability in proteins by the heat capacity change of unfolding ΔC_p_ [68,69,70].

In our analysis to elucidate the factors contributing to protein thermostability, we found some surprising results. The increase in aromaticity is beneficial to thermostability in proteins. However, we found that some thermophilic proteins, including nitrogen regulatory protein, thioredoxin, and chemotaxis protein CheW, have fewer aromaticity values than their non-thermophilic orthologous. Although chemotaxis protein CheW has a lower aromaticity value, the percentage of the secondary structures coil and sheet is higher, which may provide thermostability to this protein. Similarly, compared with their non-thermophilic orthologous, nitrogen regulatory protein has more hydrogen bonds and salt bridges, a shorter bond length, a wider DHA angle (Table 3), and a higher helix percentage, and thioredoxin has a short bond length and a higher helix structure, which may contribute to their thermostability.

We also found that thermophilic cold shock protein has no helix structure, while its non-thermophilic orthologous has 4% of the helix. However, thermophilic cold shock protein has more salt bridges, a shorter bond length, a wider DHA angle (Table 3), a higher aromaticity value, and more coil structures, which are favorable for imparting thermostability and may compensate for the absence of helix structure. Similarly, thermophilic proteins RecA, DNA-binding protein HU, and chemotaxis protein CheW also have fewer helix structures in them as compared with their non-thermophilic orthologous. However, thermophilic RecA has more hydrogen bonds, a slightly short bond length, and more coils and sheets, which may compensate for the reduction helix, thereby providing thermostability to this protein. CheW has a short bond length and a greater proportion of coil and sheet. The DNA-binding protein HU has a higher ratio of hydrogen bonds, a higher aromaticity value, and a greater proportion of coil. These factors may contribute to their thermostability.

In addition, thermophilic cold shock protein, transcription antitermination protein NusG, and the DNA binding protein HU have a lower sheet content. However, cold shock protein has more salt bridge, a shorter bond length, and a higher percentage of coil structure; transcription antitermination protein NusG has more hydrogen bond, a higher aromaticity value, more coil, and more helix structure; and the DNA binding protein HU has a higher aromaticity value and more coils in its secondary structure; all of which favor thermostability. The nitrogen regulatory protein and thioredoxin have the same proportion of sheets when compared with their non-thermophilic orthologous. This implies that sheet structure does not contribute to their thermostability. However, nitrogen regulatory protein and thioredoxin have more polar AAs, a shorter bond length, and a higher percentage of helix structure, factors which may contribute to their thermostability.

Moreover, thermophilic proteins including nitrogen regulatory protein and thioredoxin showed a slightly lower proportion of coil. However, thermophilic nitrogen regulatory protein contains more polar AAs; therefore, the number of hydrogen bonds and salt bridges is greater when compared with its non-thermophilic orthologous. These factors may lead to differences in thermostability between thermophilic nitrogen regulatory protein and its non-thermophilic orthologous. In thermophilic thioredoxin, the bond length is shortened and the proportion of helical structure is increased, which may be responsible for its thermostability.

Hydrogen bond analysis showed that thermophilic proteins, including cold shock protein, thioredoxin, chemotaxis protein CheW, and adenylate kinase, have fewer ratios of hydrogen bonds than their non-thermophilic orthologous. Although the ratio of hydrogen bonds in thermophilic proteins cold shock protein, chemotaxis protein CheW, and thioredoxin is lower, the bond length is shorter than in their non-thermophilic orthologous. It has been reported that a hydrogen bond with a shorter bond length is more stable than one with a wider bond length [71], implying that a shorter bond length may make the hydrogen bonds more stable. Hence these proteins are more thermally stable than their non-thermophilic orthologous. However, for the thermophilic adenylate kinase, not only is the ratio of hydrogen bonds small, but also the bond length is slightly greater than in their non-thermophilic orthologous, which is unfavorable for thermostability. Our analysis showed that thermophilic adenylate kinase has more helix, coil, and sheet, which may compensate for the smaller ratio of hydrogen bonds in this protein.

In salt bridge analysis, thermophilic proteins thioredoxin, adenylate kinase, RecA, and chemotaxis protein CheW showed a smaller ratio of salt bridges than their non-thermophilic orthologous. In thioredoxin, the helix structure percentage is higher than in its non-thermophilic orthologous, and in adenylate kinase, helix, coil, and sheet structure are greater; these secondary structures may be the factors leading to their thermostability. Thermophilic chemotaxis proteins CheW and RecA showed a slightly shorter bond length and an increased percentage of coil and sheet in their secondary structures than did their non-thermophilic orthologous, which may contribute to their thermostability.

## 3. Materials and Methods

### 3.1. Data Collection

Thermophilic proteins were collected from our previous study on thermophilic proteins [32]. We searched non-thermophilic orthologous using BLAST (Basic Local Alignment Search Tool). We preferred the thermophilic and non-thermophilic orthologous pairs with a high difference in optimum growth temperature (OGT), which could provide us with a clear cause for thermostability. For obtaining thermophilic and non-thermophilic protein pairs with more OGT differences, we considered thermophilic proteins with OGT > 60 °C and their non-thermophilic with OGT < 40 °C to keep the OGT difference at least 20 °C between thermophilic proteins and their non-thermophilic orthologous. As a result, 10 pairs were obtained, which were listed in Table 4. All the analyses were performed on these data. The analyses included sequence-based analysis and structure-based analysis.

### 3.2. Sequence-Based Analysis

The sequence-based analysis included the occurrence frequency of amino acids (AA) and the relative occurrence frequency of aromatic amino acids (aromaticity) [72,73,74,75,76].

#### 3.2.1. Occurrence Frequency of Amino Acids

In order to find sequence-based differences, we calculated the occurrence frequency of AA in thermophilic and non-thermophilic proteins [77]. The occurrence frequency of AA is the frequency of 20 amino acids in a protein sequence, which is given by:

(1) $$f(t)=\frac{N(t)}{N},t\in \{A,C,D,\ldots ,Y\}$$

where *f*(*t*) is the frequency of amino acid *t*, *N*(*t*) is the number of amino acid *t* present in the protein sequence, and *N* is the length of the protein sequence [78].

#### 3.2.2. Aromaticity

Aromaticity is a relative occurrence of aromatic amino acids (phenylalanine, tyrosine, and tryptophan) in a protein. The aromaticity value of a protein can be calculated by the formula given below:

(2) $$Aromaticity=\sum_{i=1}^{20}\gamma_{i}f_{i}$$

where *f_i_* represents the relative frequency of amino acid *i*, *γ_i_* is taken as 1 when the amino acid is aromatic, and *γ_i_* is taken as 0 when the amino acid is not aromatic amino acid [56].

### 3.3. Structure-Based Analysis

The structure-based analysis included the analysis of the secondary structure of proteins, namely coil, loop, helix, and turn, and the analysis of hydrogen bonds and salt bridges and their bond length and bond angle. To visualize and analyze the secondary structure of the thermophilic and non-thermophilic proteins, a discovery studio visualizer was used, and the percentages of coils, sheets, helices, and turns were calculated [79] using the following formula:

(3) $$p(sc)=\frac{nAAs(sc)}{N},sc\in \{helix,coil,sheet,coil\}$$

where *p*(*sc*) represents the percentage of secondary structure *sc*, *nAAs*(*sc*) represents the number of amino acids in secondary structure *sc*, and *N* represents the total number of amino acids.

In addition, the ratios of hydrogen bonds and salt bridges were calculated by the following formula:

(4) $$ratio=\frac{number(Ther)}{\left[number(Ther)+number(non-Ther)\right]}$$

where *number(Ther)* and *number(non-Ther)* are the numbers of hydrogen bonds or salt bridges in the thermophilic proteins and non-thermophilic proteins, respectively. Moreover, to compare the strength of hydrogen bonds and salt bridges between thermophilic and non-thermophilic proteins, the average bond angle and average bond length were also calculated.

## 4. Conclusions

Enzymes are excellent biocatalysts and have many applications in scientific research and industry. In recent years, the use of biocatalysts on an industrial scale has increased. Biocatalysts are more sustainable, efficient, selective, and less environmentally and physiologically toxic compared to traditional chemical catalysts. However, industrial processes are carried out at higher temperatures, and stability of biocatalysts at such temperatures is a major concern. The best way to solve this problem is to use enzymes produced by thermophiles or to design thermostable enzymes.

Thermophiles are organisms that can survive at elevated temperatures. Thermophiles produce thermally stable proteins. Understanding thermal stability in these proteins is essential to theoretically describe the principles behind protein thermostability as well as to design the thermostable proteins/enzymes that can meet the demand of industrial processes. To arrive at the principles behind protein thermostability, we analyzed the sequences, secondary structures, hydrogen bonds, salt bridges, bond lengths, bond angles, and aromaticity values of 10 thermophilic proteins and their non-thermophilic orthologous. The analysis shows that the frequencies of polar AAs glutamic acid, histidine, lysine, arginine, and tyrosine are higher in thermophilic proteins, which may provide thermostability to protein through the formation of hydrogen bonds, salt bridges, and other long- and short-range interactions. Moreover, the nonpolar AA proline is more common in thermophilic proteins. The rigid structure of the proline may play a role in reducing the entropy of the main chain and in resisting the protein’s unfolding. In addition, thermophilic proteins have a higher frequency of aromatic AAs which form aromatic interactions. The aromatic interactions are also valuable for providing thermostability to the proteins. In the secondary structure, the increase in the proportion of coil, helix, and sheet structure has an important contribution to thermostability in the proteins. In addition, an increased number of hydrogen bonds and salt bridges, their shorter bond length, and their wider bond angle also offer thermal stability to the protein structure. In some proteins, all the above-mentioned factors work individually, but in other proteins, these factors work together in a subtle combination. Therefore, it can be concluded that the basis of thermostability is complex and is affected by different factors alone or in different combinations.

## Figures and Tables

**Table 1:** Percentage of secondary structures in thermophilic (Ther) and non-thermophilic (non-Ther) proteins.

	Helix (%)		Sheet (%)		Coil (%)		Turn (%)	
	Ther	Non-Ther	Ther	Non-Ther	Ther	Non-Ther	Ther	Non-Ther
Hsp40 Chaperones	6.67	6.38	40.00	31.91	48.89	43.62	4.44	18.09
Nitrogen Regulatory Protein	31.58	17.86	31.58	32.14	24.21	30.36	12.63	19.64
Cold Shock Protein	0.00	4.48	45.45	50.75	45.45	29.85	9.09	14.93
DNA-Binding Protein HU	47.06	51.32	22.35	25.00	24.71	9.21	5.88	14.47
Ribosome-Binding Factor A	41.51	38.89	16.04	14.81	33.96	32.41	8.49	13.89
NusG	19.21	0.00	24.86	43.55	37.29	35.48	18.64	20.97
Protein RecA	38.30	46.82	20.18	16.76	33.04	26.88	8.84	9.54
Thioredoxin	31.43	29.31	21.90	22.41	22.86	26.72	23.81	21.55
CheW	9.27	13.17	37.75	34.13	42.38	36.53	10.60	16.17
Adenylate Kinase	52.22	49.07	14.78	14.49	22.66	22.43	10.34	14.02

**Table 2:** The ratio of hydrogen bonds and salt bridges in thermophilic protein.

Proteins	Hydrogen Bond Ratio	Salt Bridge Ratio
Hsp40 Chaperones	0.50	0.65
Nitrogen Regulatory Protein	0.70	0.63
Cold Shock Protein	0.44	1.00
DNA-Binding Protein HU	0.52	0.86
Ribosome-Binding Factor A	0.55	1.00
NusG	0.55	0.60
Protein RecA	0.66	0.43
Thioredoxin	0.48	0.31
CheW	0.38	0.25
Adenylate Kinase	0.48	0.40

**Table 3:** Average bend length and DHA angle in thermophilic and non-thermophilic proteins.

Proteins	Average Bond Length (Å)		Average DHA Angle	
	Thermophilic	Non-Thermophilic	Thermophilic	Non-Thermophilic
Hsp40 Chaperones	2.41	2.40	135.20	134.41
Nitrogen Regulatory Protein	2.94	3.08	109.53	106.45
Cold Shock Protein	2.25	2.99	135.85	108.01
DNA-Binding Protein HU	3.00	3.00	108.30	109.00
Ribosome-Binding Factor A	2.36	2.21	139.29	134.75
NusG	2.34	2.34	135.82	136.37
Protein RecA	3.02	3.04	109.23	108.02
Thioredoxin	2.24	2.31	141.92	142.46
CheW	2.30	2.36	133.03	142.06
Adenylate Kinase	3.04	3.05	109.44	108.51

**Table 4:** Thermophilic proteins and their non-thermophilic orthologous.

Protein Name	PDB ID	Organism Name	OGT
Hsp40 chaperones	6PRP	*Thermus thermophilus*	80
Hsp40 chaperones	6PQM	*Escherichia coli*	37
Nitrogen regulatory protein	2EG1	*Aquifex aeolicus*	85
Nitrogen regulatory protein	1PIL	*Escherichia coli*	37
Cold shock protein	1G6P	*Thermotoga maritima*	80
Cold shock protein	1CSP	*Bacillus subtilis*	25–35
DNA-binding protein HU	5EKA	*Thermus thermophilus*	85
DNA-binding protein HU	1MUL	*Escherichia coli*	37
Ribosome-binding factor A	2KZF	*Thermotoga maritima*	90
Ribosome-binding factor A	1KKG	*Escherichia coli*	37
Transcription antitermination protein NusG	2LQ8	*Thermotoga maritima*	80
Transcription antitermination protein NusG	2MI6	*Mycobacterium tuberculosis*	30–32
Protein RecA	3HR8	*Thermotoga maritima*	80
Protein RecA	4OQF	*Mycobacterium tuberculosis*	32
Thioredoxin	1RQM	*Alicyclobacillus acidocaldariu*	60–65
Thioredoxin	2L4Q	*Mycobacterium tuberculosis*	30–32
Chemotaxis protein CheW	1K0S	*Thermotoga maritima*	80
Chemotaxis protein CheW	2HO9	*Escherichia coli*	37
Adenylate kinase	2RGX	*Aquifex aeolicus*	85
Adenylate kinase	4K46	*Photobacterium profundum*	15

## Data Availability

Not applicable.
